# Supplementary figures and images for: A data-driven approach for constructing mutation categories for mutational signature analysis
Source: PLoS Comput Biol. 2021 Oct 19;17(10):e1009542. doi: 10.1371/journal.pcbi.1009542 (PMC8555780; doi:10.1371/journal.pcbi.1009542)

BRCA\_TEST (k\_cosmic=12)

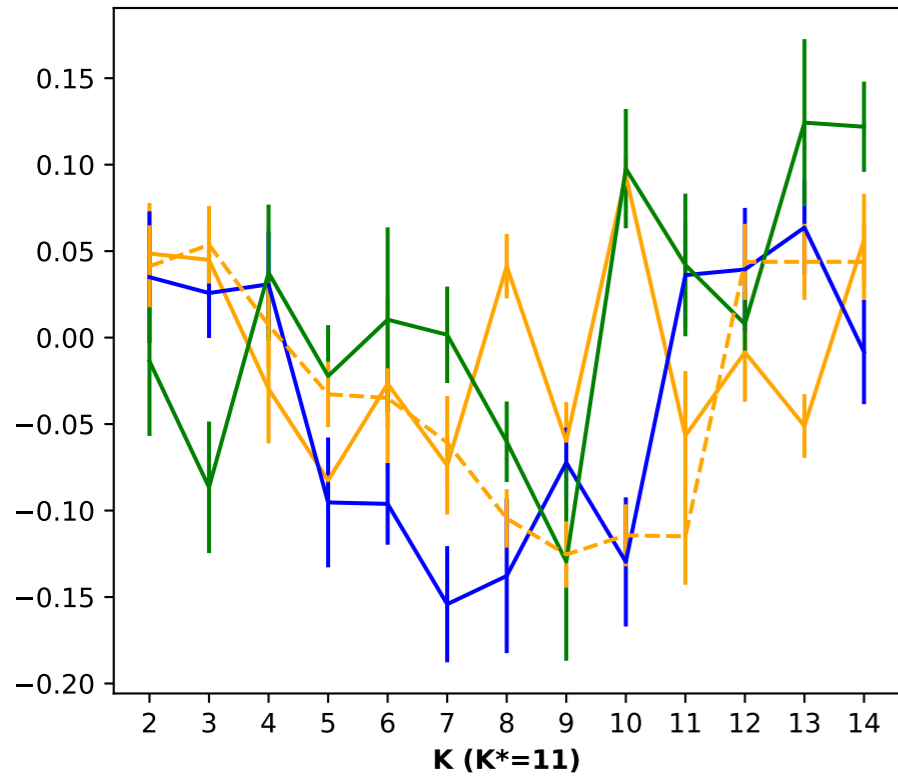

PRAD\_TEST (k\_cosmic=3)

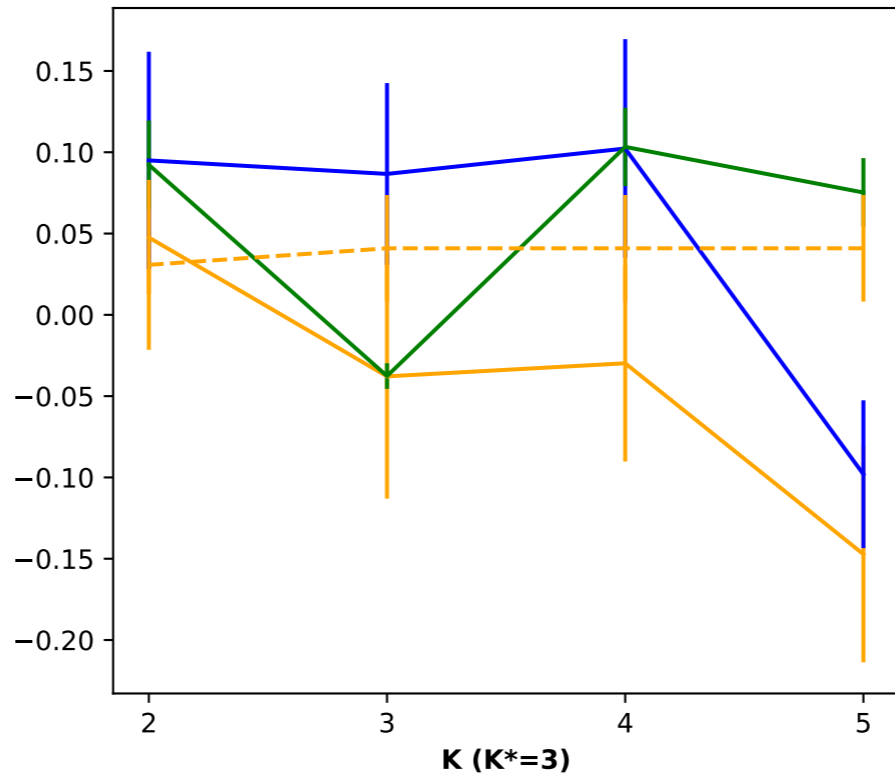

OV (k\_cosmic=3)

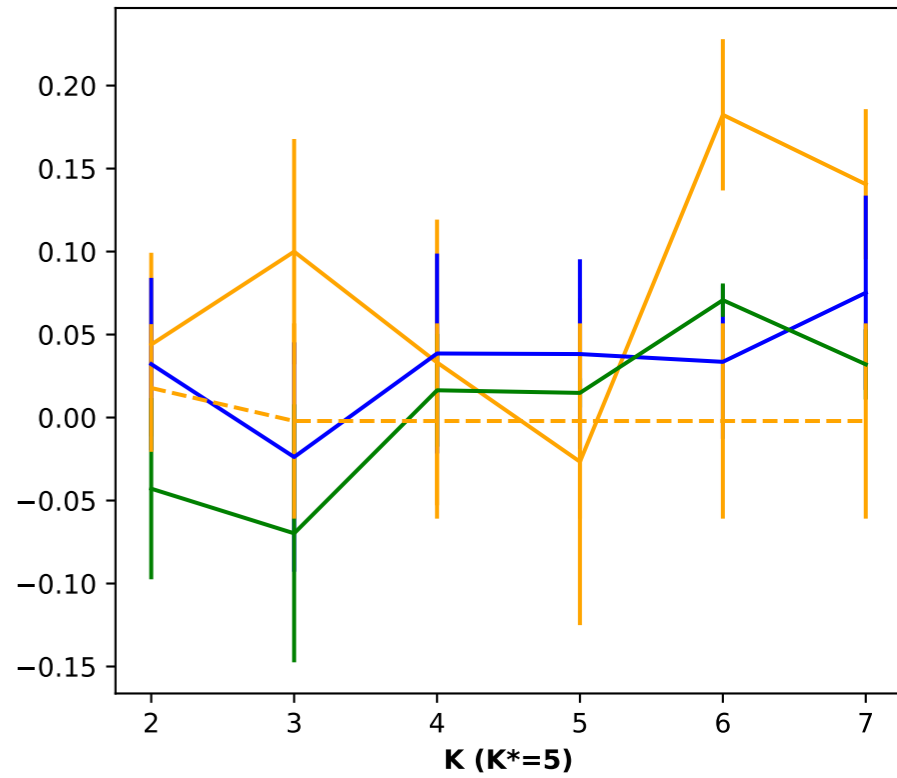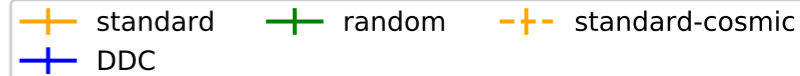

Supplement: S2 Fig — For each K in the range of number of components [2, max(k_cosmic, K*) + 2], we apply NMF to the WGS samples to learn the signature matrix H and then derive the exposure matrix W from the WES samples using NNLS. We learn the CCA coefficients using WES training samples and compute the resulting correlation on the test samples. The reported correlation is the average over 10-fold cross validation. Error bars represent the standard deviation of multiple evaluation runs. (PDF) [file pcbi.1009542.s002.pdf]

BRCA\_TEST (k\_cosmic=12)

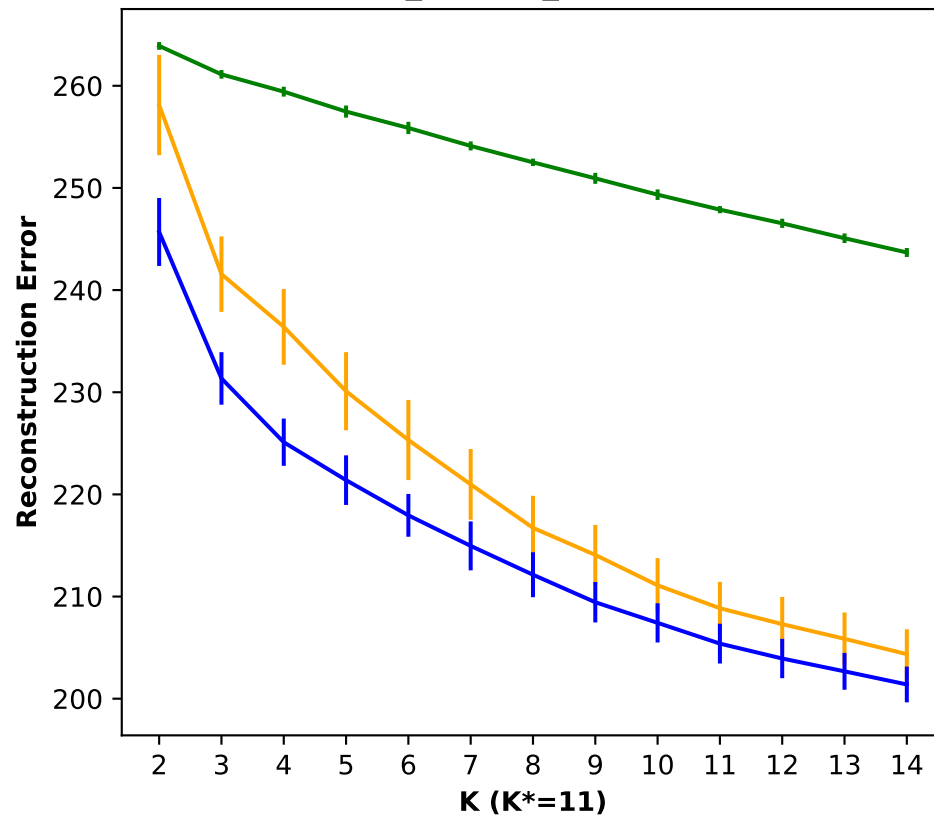

PRAD\_TEST (k\_cosmic=3)

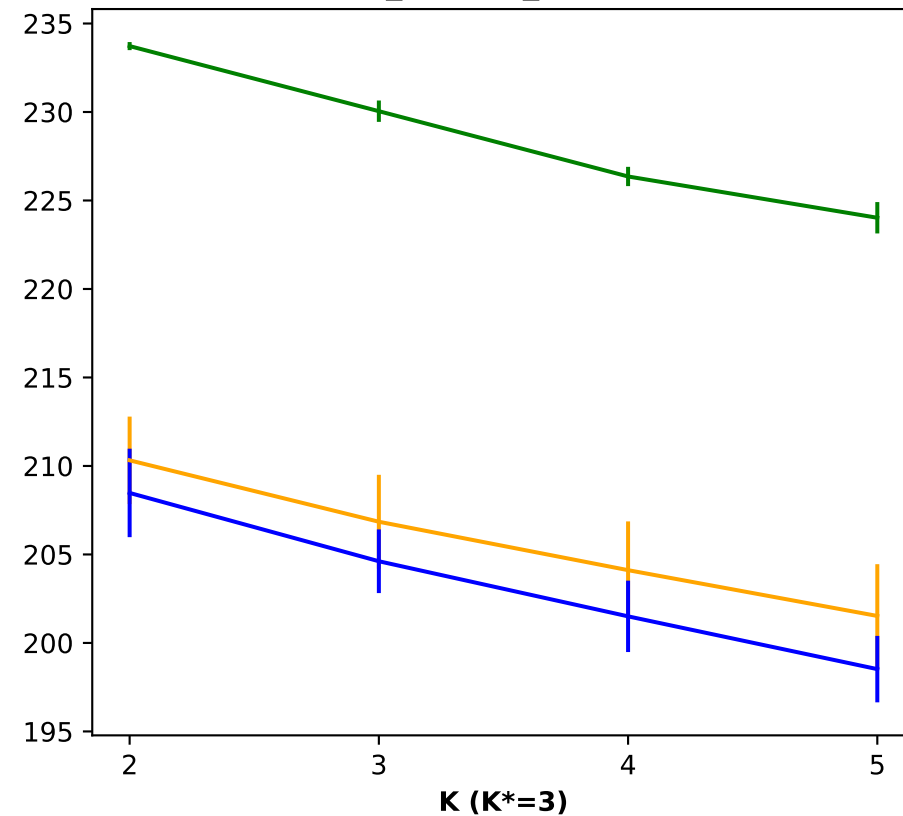

OV (k\_cosmic=3)

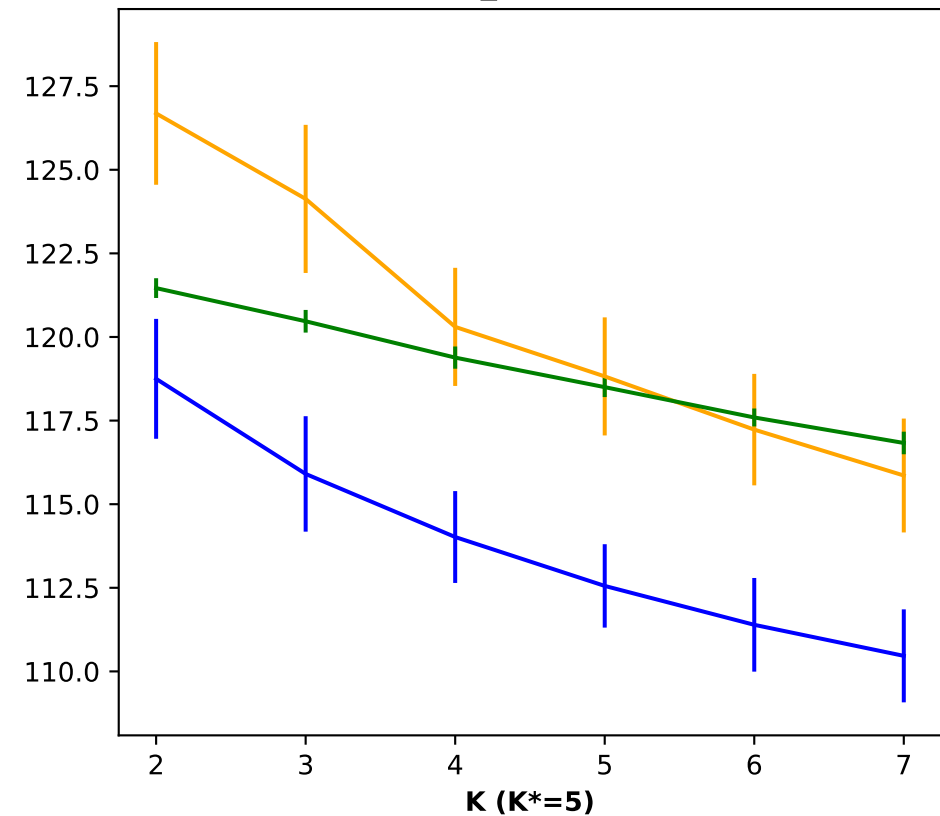

standard DDC random

Supplement: S3 Fig — For each K in the range of number of components [2, max(k_cosmic, K*) + 2], we apply NMF to the WGS samples to learn the signature matrix H and then derive the exposure matrix W from the test samples using NNLS. The reconstruction error (Kullback–Leibler divergence) is the approximation error of this factorization with respect to the test samples of the (normalized) count matrix V. NMF is applied 10 times and the reported reconstruction error is the average over these runs. Error bars represent the standard deviation. (PDF) [file pcbi.1009542.s003.pdf]

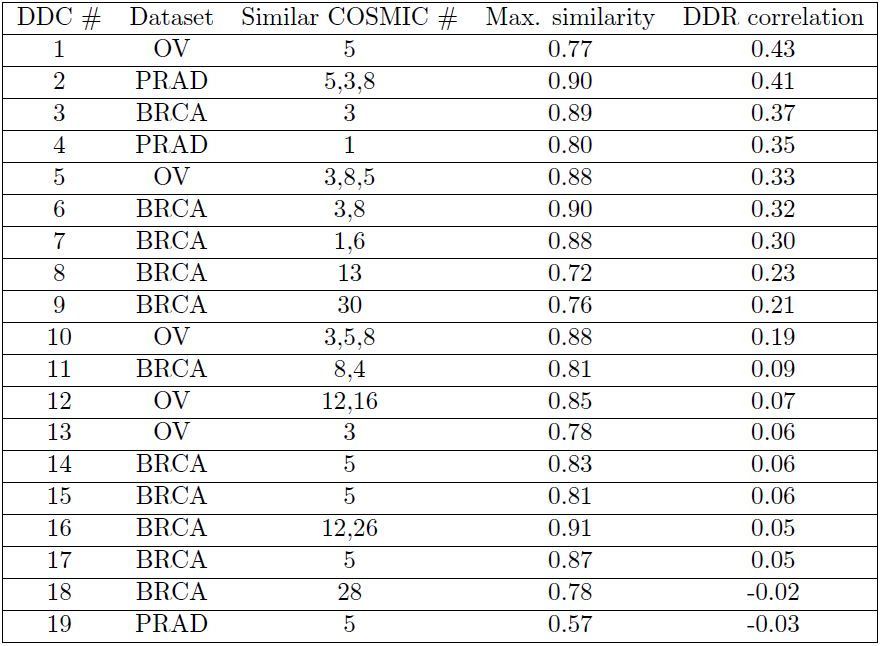

Supplement: S1 Table — The signatures are ordered according to their correlation with the expression of DDR genes. For each signature, given are also the dataset that it was derived from, its similar COSMIC signatures (ordered, cosine similarity > 0.8) and the similarity to the most similar COSMIC signature. In case there is no COSMIC signature with cosine similarity > 0.8 to the DDC signature, we report the COSMIC signature with the greatest similarity to it. (JPG) [file pcbi.1009542.s004.jpg]
