# Supplementary material for: A data-driven approach for constructing mutation categories for mutational signature analysis
Source: PLoS Comput Biol. 2021 Oct 19;17(10):e1009542. doi: 10.1371/journal.pcbi.1009542 (PMC8555780; doi:10.1371/journal.pcbi.1009542)
Supplement: S1 Fig — The signature is depicted using both the standard categories (top) and the DDC ones (bottom, categories presented using the IUPAC code). This signature is similar to COSMIC Signatures 1 and 6 (cosine similarity 0.88 and 0.84 respectively), and its exposure is correlated with the expression of DDR genes (Pearson correlation 0.30). (PDF) [file pcbi.1009542.s001.pdf]

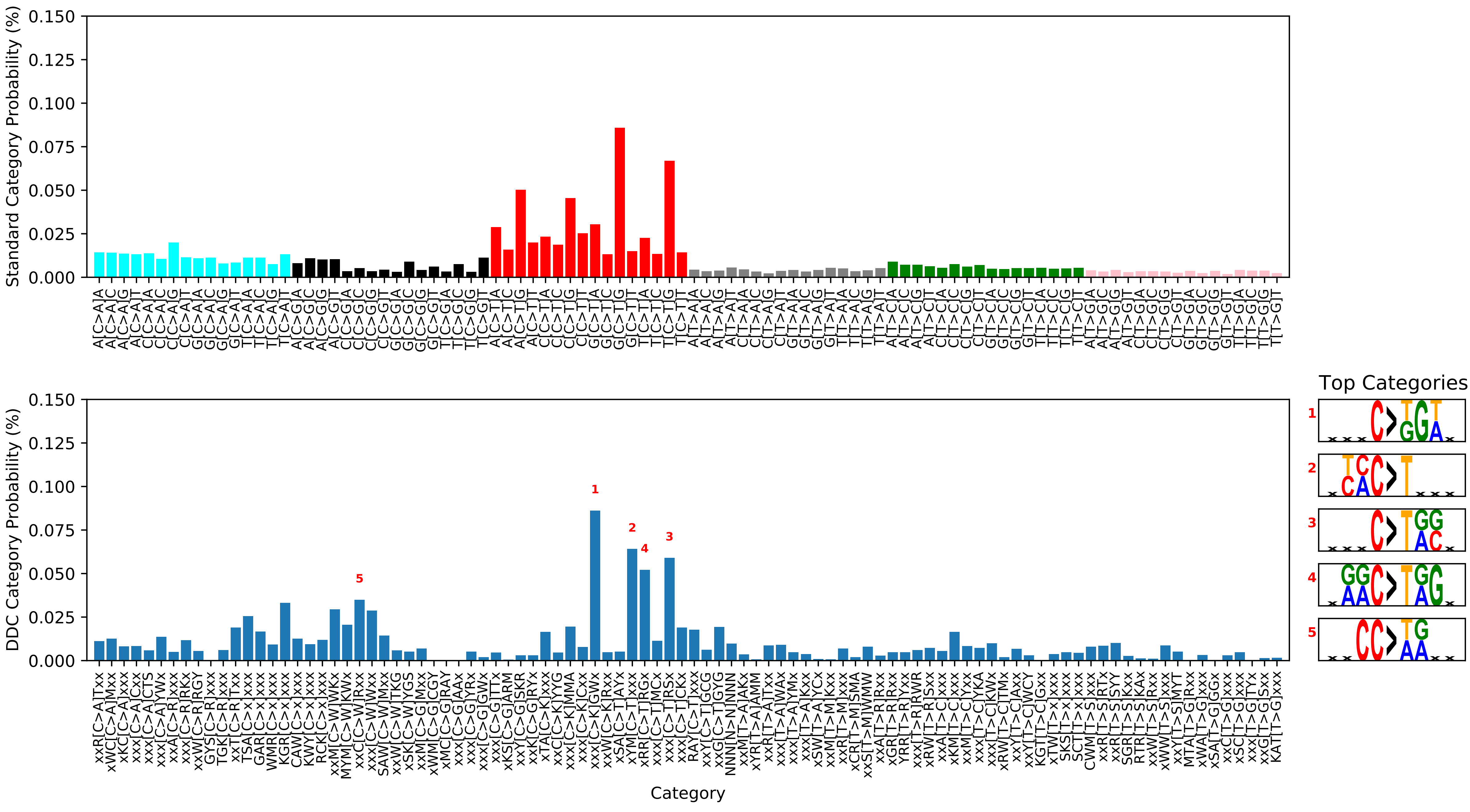

Standard Category Probability (%)
0.150
0.125
0.100
0.075
0.050
0.025
0.000
A[C>A]A A[C>A]C A[C>A]G A[C>A]T C[C>A]A C[C>A]C C[C>A]G C[C>A]T G[C>A]A G[C>A]C G[C>A]G G[C>A]T T[C>A]A T[C>A]C T[C>A]G T[C>A]T
A[C>G]A A[C>G]C A[C>G]G A[C>G]T C[C>G]A C[C>G]C C[C>G]G C[C>G]T G[C>G]A G[C>G]C G[C>G]G G[C>G]T T[C>G]A T[C>G]C T[C>G]G T[C>G]T
A[C>T]A A[C>T]C A[C>T]G A[C>T]T C[C>T]A C[C>T]C C[C>T]G C[C>T]T G[C>T]A G[C>T]C G[C>T]G G[C>T]T T[C>T]A T[C>T]C T[C>T]G T[C>T]T
A[T>A]A A[T>A]C A[T>A]G A[T>A]T C[T>A]A C[T>A]C C[T>A]G C[T>A]T G[T>A]A G[T>A]C G[T>A]G G[T>A]T T[T>A]A T[T>A]C T[T>A]G T[T>A]T
A[T>C]A A[T>C]C A[T>C]G A[T>C]T C[T>C]A C[T>C]C C[T>C]G C[T>C]T G[T>C]A G[T>C]C G[T>C]G G[T>C]T T[T>C]A T[T>C]C T[T>C]G T[T>C]T
A[T>G]A A[T>G]C A[T>G]G A[T>G]T C[T>G]A C[T>G]C C[T>G]G C[T>G]T G[T>G]A G[T>G]C G[T>G]G G[T>G]T T[T>G]A T[T>G]C T[T>G]G T[T>G]T
DDC Category Probability (%)
0.150
0.125
0.100
0.075
0.050
0.025
0.000
1
2
4
3
5
xxR[C>A]Txx xWC[C>A]Mxx xKC[C>A]xxx xxx[C>A]Cxx xxx[C>A]CTS xxx[C>A]YWx xxA[C>R]xxx xxx[C>R]RKx xxW[C>R]RGY GYS[C>R]xxx TGK[C>R]Txx xxT[C>R]Txx TSA[C>x]xxx GAR[C>x]xxx WMR[C>x]xxx KGR[C>x]xxx CAW[C>x]xxx KWY[C>x]xxx RCK[C>x]xxx
xxM[C>W]WKx MYM[C>W]KWx xxC[C>W]Rxx xxx[C>W]Wxx SAW[C>W]Mxx xxW[C>W]TKG xSK[C>W]YGS xxM[C>G]Mxx xWM[C>G]CGY xMC[C>G]RAY xxx[C>G]AAx xxx[C>G]YRx xxx[C>G]GWx xxx[C>G]TTx xKS[C>G]ARM xxY[C>G]SKR xxK[C>G]RYx
xTA[C>K]xxx xxC[C>K]YYG xxx[C>K]MMA xxx[C>K]Cxx xxx[C>K]GWx xxW[C>K]Rxx xSA[C>T]AYx xYM[C>T]xxx xRR[C>T]RGx xxx[C>T]MCx xxx[C>T]RSx xxx[C>T]CKx RAY[C>T]xxx xxY[C>T]GCG xxG[C>T]GYG NNN[N>N]NNN
xxM[T>A]AKx xYR[T>A]AMM xxR[T>A]Txx xxx[T>A]WAx xxx[T>A]YMx xxx[T>A]Kxx xSW[T>A]YCx xxM[T>M]Kxx xxR[T>M]xxx xCR[T>M]SMA xxS[T>M]WMW xxA[T>R]Rxx xGR[T>R]Rxx YRR[T>R]Yxx xxx[T>R]RWR xRW[T>R]Sxx
xxA[T>C]xxx xKM[T>C]xxx xxM[T>C]Yxx xxx[T>C]YKA xxx[T>C]KWx xRW[T>C]TMx xxY[T>C]Axx xxY[T>C]WCY KGT[T>C]Gxx xTW[T>x]xxx SKS[T>x]xxx SCT[T>x]xxx
CWM[T>S]xxx xxR[T>S]RTx xxR[T>S]SYY SGR[T>S]Kxx RTR[T>S]KAx xxW[T>S]Rxx xWW[T>S]xxx xxY[T>S]MYT MTA[T>G]Rxx xWA[T>G]xxx xSA[T>G]GGx xxC[T>G]xxx xSC[T>G]xxx xxx[T>G]TYx xxG[T>G]Sxx KAT[T>G]xxx
Category
Top Categories
1
2
3
4
5
