# Supplementary material for: A data-driven approach for constructing mutation categories for mutational signature analysis
Source: PLoS Comput Biol. 2021 Oct 19;17(10):e1009542. doi: 10.1371/journal.pcbi.1009542 (PMC8555780; doi:10.1371/journal.pcbi.1009542)
Supplement: Attachment — Submitted filename: Response to reviewers.docx [file pcbi.1009542.s005.docx]

Dear Editor,

Please find enclosed a revision of our paper "A data-driven approach for constructing mutation categories for mutational signature analysis". We have revised our manuscript according to the referees' comments, a point-by-point response appears below.

Very truly yours,

Roded Sharan

Reviewer #1:

• The method employs a genetic algorithm that is based on crossover and mutation. It is unclear to me what relevance/interpretation the crossover would have in this context, given that we are not talking about sexual reproduction in the context of tumour cell proliferation. I understand the “parents” in this case are abstracted to refer to different mutation categories, but why would there be a crossover in the process of cell division?

We now provide a reference to genetic algorithms which represent a common class of methods for hard optimization problems that are inspired by biology. The role of crossover in genetic algorithms is to combine information from two “parents” (solutions from the previous generation) to create a new solution. Although this process is inspired by crossover that happens during sexual reproduction, it is used in genetic algorithms merely as an operator that allows for possible creation of diverse solutions with potentially higher fit compared to the previous generation.

• Why 96 categories? This is not explained, and I cannot think of why it may be beneficial to replicate the 96-category standard given that the categories are being changed.

As mentioned in the manuscript, we use 96 categories for ease of comparison to standard approaches that are based on 96 categories. Importantly, this eliminates the number of categories in a categorization as a possible explanation for differences in our results versus standard approaches. We now mention that also in the Conclusions and suggest running the method with flexible number of categories for future research.

• It is somewhat unclear what is going on when exposures and expression are being correlated. Are there any correlation cut-offs applied to guide signature selection? These correlations should also be plotted/reported, as they would enhance the interpretability of the procedure.

There is no correlation-based signature selection. Signatures are extracted using NMF and all corresponding exposures are correlated with expression data (Figure 4).

• For the calculation of the reconstruction error, 10 runs with randomly initialised W,H are performed and the smallest error obtained is reported. It may be fairer to report the mean and confidence interval instead – and this could be updated in Fig 3.

We perform multiple initializations and report the smallest error to overcome the tendency of NMF to converge to a local optimum. As suggested by the reviewer we added a supplementary figure (Figure 8) with the mean and std to provide complete information on the process, although we believe that the minimum is the more informative measure in this case.

• Figure 4: DDC improvement is very marginal in BRCA - any particular interpretation as to why this is the case?

We believe that this stems from the difference in number of signatures and their DDR association between the cancer types. Specifically, BRCA has 12 associated signatures, only 6-8 of which are known to be associated with DDR, while both OV and PRAD have only 3 signatures and 2/3 have some association with DDR (Sig 3 and 5 for OV and Sig 5 and 6 for PRAD). We added this explanation to the Results section.

• Table 1: it would be useful to discuss further the fact that more often than not the most correlated subset and proposed aetiology do not match. I acknowledge part of this is covered in the discussion already (BER and deamination connection), but it may also be worth considering that 4/7 top signatures are essentially flat signatures. Would there be a tendency for flat signatures to naturally display the highest correlations? Is Pearson’s correlation the best measure of correlation, particularly when some contributions will be so small?

Since we perform multi-objective optimization (more than one cancer type) to optimize the correlation between signature exposures and DDR expression, and find a categorization that generalizes to these cancer types, it is not surprising that signatures 1,3,5,8 (among them the ‘flat’ 3,5,8) that are active in these cancer types are also showing high correlations. We should also consider that flat signatures are more likely to come up as COSMIC signatures that are similar to ‘our’ signatures in cases where ‘our’ signature is novel. The top signature is not similar (cosine < 0.8) to any COSMIC signature. We already discuss the relationship between the proposed etiologies and the most correlated DDR subset. In addition, we now report also the most correlated DDR subsets to the corresponding COSMIC signatures (and find that these are consistent with ‘our’ most correlated DDR subsets; Table 1).

• In addition to this, it may be useful to explore further, or at least display, some of the non-flat signatures - possibly a signature correlated with an MMR-deficiency signature like SBS6?

As requested, we added a figure (Figure 6) displaying this signature to the supplement.

• All signatures derived in the analysed cohorts should be reported, along with the corresponding DDR correlations.

We added a supplementary table (Table 2) with the full information.

• Do the signatures uncovered make sense in the context of the biology of the three diseases studied, and that of other reports from the literature? Are there any new biological insights that this analysis brings?

Beside the signatures similar to 1,3,5,8 that are in Table 1 and do appear in these cancer types, we now discuss also the rest of the extracted signatures.

• The authors should expand on the motivation, interpretability and applicability of their newly uncovered signatures. Can these signatures improve DDR deficiency prediction? Other benefits and future directions should be specified.

We added a discussion about these points to the Conclusions and mentioned the clinical potential of the new signatures.

Reviewer #2:

The authors state that their algorithm took 2 days to run on a 40-core server, and the description of the tuning procedure indicates that they ran a total of 4+3+3+4=14 iterations to explore different values of the model parameters, which suggests the tuning process took 4 weeks of compute time. I appreciate that this is a new approach to mutation signature analysis and optimization is not necessarily a priority, but I’m not convinced that the novelty of the findings justifies this massive computational burden. Given the length of the paper and focus on describing the algorithm, I reviewed this primarily as a method/software paper, and as such, I would expect the software to have a runtime at least on the same order of magnitude as existing methods (The authors state that they identified a categorization that generalizes across datasets [line 320], so at the very least I would expect these data to be made publicly available so other users can treat it as a “pre-trained” model).

The runtime of the method (2 days for a given parameter set) should not be compared to existing methods, simply because there are no current methods (to the best of our knowledge) that find an alternative mutation categorization. The method should be treated as a pre-step for mutational signature analysis that is performed only rarely when the mutation data base is updated, hence runtime should not be a consideration. We added this point to the discussion and also provided the categorization we have computed in the GitHub.

On the other hand, if this is meant to be more of a standalone research paper, the biological insights lack novelty/depth. For example, Table 1 indicates DDC signature 1 is the only signature without a similar COSMIC signature or proposed etiology, but is most correlated to NER genes and mildly correlated with age. How do we know this is not just an artifact of fitting to the data or a spurious correlation? Is there any evidence in prior literature that NER genes or age-related processes are enriched for C>T transitions in the G downstream context (line 407)?

This is indeed a method paper, and an in-depth, large scale mutational signature analysis across many samples and tumor types could be performed in the future to extract a full catalog of mutational signatures that are based upon the optimized categorization. Regarding DDC signature 1, as we also hint to in the text, we believe that it could be in fact similar to COSMIC signature 1, even though its cosine similarity to it (0.77) is slightly below the threshold we used, since: (1) COSMIC signature 1 is also enriched with C>T substitutions in the G downstream context and known to be age-related; and (2) we found that the activity of COSMIC signature 1 in ovarian cancer samples - the cancer type from which DDC signature 1 was extracted – is also most correlated to NER genes.

It would also be nice to include:

- A supplementary figure comparing performance across the different parameter values evaluated (similar to Figs 3 & 4 but comparing e.g., q = [0, 0.4, 0.8, 1])

Our trials with the four parameters were performed in order to calibrate the fitness optimization process carried out by the genetic algorithm. This parameter tuning procedure is standard for genetic algorithms and allows better optimization by avoiding premature convergence, providing sufficient diversity in the population, etc. Below is a figure comparing between different values of q (crossover rate) – with fixed values for the other parameters - that demonstrates how the q values affect the optimization throughout the generations. For each parameter, we picked the value that leads to better optimization. We chose not to add these figures to the paper.

- scripts in the Github repository so users can compare performance of DDC on their data against standard approaches

We added a comparison script (categorization_evaluation.py) to GitHub.

Line 303-307: The authors state that they selected COSMIC signatures only found to be active in the investigated cancer types. It would be good to include some supplementary results comparing the DDC signatures against all COSMIC signatures to affirm that the matching signatures in Table 1 are not just reflecting a confirmation bias. Moreover, the COSMIC signatures believed to be associated with sequencing artifacts should absolutely be considered in this analysis (https://cancer.sanger.ac.uk/signatures/sbs/).

We did compare the signatures in the table to all 30 COSMIC v2 signatures (now clarified) and reported ones with similarity > .8 (Table 1). The COSMIC signatures believed to be associated with sequencing artifacts are reported only on v3. We have now added these to the comparison (results remain unchanged).

The authors state that their choice to collapse longer sequence contexts into the 96 most informative categories was solely for the sake of comparing their findings directly with the standard 96-subtype approaches, not by any biological motivation. It would be nice to see a more thorough analysis of how many categories are actually needed--what is the minimal number of categories required to match the reconstruction error of standard approaches? How much is reconstruction error reduced by increasing the number of categories? Do the identified categories remain fairly stable?

Indeed, this is an interesting area for future research, and we discuss it now in detail. See answer to similar comment (#2) by Reviewer 1.

I had some difficulty getting the DDC software to run. It would help to include a conda/pip environment config file to ensure users are always using tested versions of the required Python libraries (using a fresh install of Miniconda, I was able to run the software after installing the scikit-learn, matplotlib, pandas, and pyrcca packages). I also got several numpy runtime warnings while running DDC, but it was not obvious if this was informational or an error that needed to be debugged (e.g., “python3.9/site-packages/numpy/lib/function_base.py:380: RuntimeWarning: Mean of empty slice.”). To run the software with the included example data, I had to create a new directory called “categorization_related_data”, move the example files to this directory, and rename them to remove underscores in the file names, because the “--datatype” parameter treats underscores as a delimiter. The code is cleanly written and easy to follow, but additional testing and documentation would go a long way towards making this software functional.

Fixed.

MINOR COMMENTS

Line 35-39--this sentence should include some references to the initial implementations of NMF-based mutation signature analysis using 96 categories (Nik-Zainal et al. 2012 [https://www.sciencedirect.com/science/article/pii/S0092867412005284] and Alexandrov et al. 2013 [https://www.nature.com/articles/nature12477])

We added the references as suggested.

Line 75, Line 168, Fig. 1--the terminology of “8-long sequence” is very confusing. Functionally, the authors are describing how they encode a 7-mer mutation subtype as an 8-character string (e.g., TGT[C>T]AAC becomes TGTCTAAC), but calling the latter a “sequence” implies they are looking at an actual 8bp DNA sequence motif, which is misleading. It would be clearer to describe the mutation categories as representing a possible 7-mer mutation subtype and us standard IUPAC codes (http://www.bioinformatics.org/sms/iupac.html) to represent “wildcards” present in the categories, e.g., NNTC(A/G)GAN would be better represented as NNT[C>R]GAN, where R represents an A or a G. This way, all categories are consistently written in a way that indicates the unmutated flanking bases and the specific mutation at the 4th position. (I’ll also note that the authors already use the “7-mer” terminology on the README of their Github repository, and the python code is already using IUPAC notation for ambiguous bases, but the --kmer parameter in the program is inconsistent with this).

We thank the reviewer for this suggestion to clarify the terminology, which we now follow.

Fig. 5--the DDC signature panel would be easier to interpret if the signature barplots indicated which bars correspond to each of the “Top Categories” in the logo plots, e.g., with a number 1-5 above the corresponding bar or color code for each category.

We added the requested information to the figures (Figures 5 and 6).

Missing code for comparing against COSMIC signatures; missing data for the DDC categories & resulting signatures

Comparison script (categorization_evaluation.py), DDC categories and signatures are now in Github.

Reviewer #3:

I do not have any major request for edit. As a minor, at the beginning of Section 2 it would be good to mention, as in line 185, that the focus on 96 categories is to compare with the standard categorization. Moreover, do the author have any thoughts on what would be the best choice of the number of categories? This could be added to Conclusions.

Indeed, see answer to similar comment (#2) by Reviewer 1.
